# Repeated exposure to kairomone-containing coffee odor improves abnormal olfactory behaviors in heterozygous oxytocin receptor knock-in mice

**DOI:** 10.3389/fnbeh.2022.983421

**Published:** 2023-02-01

**Authors:** Kazumi Osada, Riyuki Kujirai, Akira Hosono, Masato Tsuda, Motoko Ohata, Tohru Ohta, Katsuhiko Nishimori

**Affiliations:** ^1^Department of Food Bioscience and Biotechnology, College of Bioresource Sciences, Nihon University, Fujisawa, Japan; ^2^The Research Institute of Health Science, Health Sciences University of Hokkaido, Tobetsu, Japan; ^3^Department of Molecular and Cell Biology, Graduate School of Agricultural Science, Tohoku University, Sendai, Japan

**Keywords:** olfactory behavior, oxytocin receptor, avoidance, Y-maze, rescued abnormality

## Abstract

The oxytocin receptor (OXTR) knockout mouse is a model of autism spectrum disorder, characterized by abnormalities in social and olfactory behaviors and learning. Previously, we demonstrated that OXTR plays a crucial role in regulating aversive olfactory behavior to butyric acid odor. In this study, we attempted to determine whether coffee aroma affects the abnormal olfactory behavior of OXTR-Venus knock-in heterozygous mice [heterozygous OXTR (±) mice] using a set of behavioral and molecular experiments. Four-week repeated exposures of heterozygous OXTR (±) mice to coffee odor, containing three kairomone alkylpyrazines, rescued the abnormal olfactory behaviors compared with non-exposed wild-type or heterozygous OXTR (±) mice. Increased *Oxtr* mRNA expression in the olfactory bulb and amygdala coincided with the rescue of abnormal olfactory behaviors. In addition, despite containing the kairomone compounds, both the wild-type and heterozygous OXTR (±) mice exhibited a preference for the coffee odor and exhibited no stress-like increase in the corticotropin-releasing hormone, instead of a kairomone-associated avoidance response. The repeated exposures to the coffee odor did not change oxytocin and estrogen synthetase/receptors as a regulator of the gonadotropic hormone. These data suggest that the rescue of abnormal olfactory behaviors in heterozygous OXTR (±) mice is due to the coffee odor exposure-induced OXTR expression.

## Introduction

Oxytocin (OXT) is a nonapeptide hormone synthesized in the paraventricular and supraoptic nuclei of the hypothalamus (Gainer, 1998) and secreted from the posterior pituitary gland. In addition to its classic effects on milk release and ejection, the OXT receptor (OXTR) system plays important roles in social behaviors (Takayanagi et al., 2005; Janak and Tye, 2015; Althammer et al., 2018) and learned behavioral patterns (Sala et al., 2011). In addition, OXT modulates context-dependent social olfactory behaviors (Choe et al., 2015; Oettl et al., 2016). Mice with a conditional OXTR gene deletion in the anterior olfactory nucleus show a deficit in social odor recognition of same-sex conspecifics (Oettl et al., 2016). In addition, OXTR signaling is crucial for odor synchronization to social cues in rats (Choe et al., 2015).

Autism spectrum disorder (ASD) is characterized by a persistent deficiency in the ability to initiate and sustain reciprocal social interaction and social communication and by a range of restricted, repetitive, and inflexible behavior patterns, interests, or activities that are clearly atypical or excessive for an individual’s age and sociocultural context (The World Health Organization’s International Classification of Diseases [11th Revision] ICD-11, 2022). ASD is a neurodevelopmental disorder group with highly variable symptoms. ASD’s genetic architecture is complex, diverse, and context-dependent, highlighting a need to study the interplay between different genetic variant types and identify genetic and non-genetic factors influencing their penetrance (Havdahl et al., 2021).

In addition to the aforementioned social abnormality, ASD symptoms may include atypical olfactory behavior and function caused by hyper- or hypo-reactivity to sensory stimuli, such as light, odors, and pain (The World Health Organization’s International Classification of Diseases [11th Revision] ICD-11, 2022). Other symptoms include aberrant sniffing regardless of odor valence (Rozenkrantz et al., 2015), an incorrect odor intensity judgment (Wicker et al., 2016), and a higher odor threshold to volatile esters (Kumazaki et al., 2016).

Oxytocin receptor-null mice have been considered an ASD model (Insel, 2010), demonstrating sociability impairment, preference for social novelty, and resistance to change in a learned behavior pattern (Sala et al., 2011). OXTR-Venus knock-in [homozygous OXTR (–/–)] mice demonstrated an acute olfactory response dysfunction to an unpleasant odor of butyric acid at the first presentation (failure of avoidance from the unpleasant odor) as a resistance to change and then persistent avoidance of later butyric acid odor presentations (Osada et al., 2018). However, dissimilar to the ASD symptoms, homozygous OXTR (–/–) mice showed elevated aggressive behavior (Takayanagi et al., 2005). Here, heterozygous OXTR (±) mice were considered mild ASD model cases for testing therapeutic methods based on previously reported observations that heterozygous OXTR (±) mice also failed to avoid the unpleasant butyric acid odor at the first presentation (Osada et al., 2018) but had no elevated aggressive behavior compared with wild-type mice (Sala et al., 2013).

The present study considers an intermediate effect of heterozygous OXTR deficiency on the olfactory behavioral disorders and preferred odor exposure-induced expression of genes that control brain functions (Yamamoto et al., 2013; Yoshida et al., 2017; Yokoyama et al., 2020). We attempted to rescue a dysfunction of the heterozygous OXTR (±) mice in acute olfactory responses to the unpleasant odor by repeated odor exposure. The repeated exposure of heterozygous OXTR (±) mice to a preferable food odor for 2–4 weeks was hypothesized to induce OXTR expression, then alter brain function and rescue abnormal olfactory behaviors. As a food odor, a deeply roasted coffee odor was chosen, which contained three kairomone compounds of the common gray wolf (*Canis lupus*): 2,6-dimethylpyrazine; 2,3,5-trimethylpyrazine; and 3-ethyl-2,5-dimethylpyrazine (Osada et al., 2013) and many preferable compounds, such as 2-methylbutanal (almond); 2,4,5-trymethyl-1,3-oxazole (aged cheeses); heptan-2-ol (coconut); 2,5-dimethylpyrazine (roasted nut); 5-methylfuran-2-carbaldehyde (almond and caramel); octanoic acid (cheese); 2-methylsulfanylfuran (coffee); and others (Mahmud et al., 2022). Although robust odors of kairomones are normally perceived as aversive and/or unpleasant odors (Osada et al., 2015), weak kairomone odors are expected to induce moderate facilitation of avoidance signaling without marked stress responses that would be alleviated by the other ingredients’ preferable odors, as observed in a previous study that used a mixture of rose odor and fox-characteristic 2,5-dihydro-2,4,5-trimethylthiazoline (TMT) odor (Matsukawa et al., 2011). In addition, some kairomones and those of the analogs contained in food induce a physiological relaxing effect (2,3-dimethylpyrazine) (Ohata et al., 2020) and protect the heart from ischemia/reperfusion injury (2-methyl-2-thiazoline) (Nishi et al., 2022). In fact, the coffee scent may relieve stresses in mice by inducing gene expression and proteins suppressed by a stressor (Yamato et al., 2002; Seo et al., 2008). Moreover, free-feeding rats prefer coffee odor over several other attractive food scents, such as cheese and orange (Tabuchi et al., 1991), suggesting that coffee odor may induce physiologically positive effects in the rat brain. For a reason stated earlier, we hypothesized that repeated coffee odor exposure could rescue a dysfunction of the heterozygous OXTR (±) mice in acute olfactory responses to the unpleasant odor.

Previously, several OXTR expression inducers have been reported. For example, binding estrogen to ER-α (Choleris et al., 2003, 2012), progesterone (Schumacher et al., 1990), interleukin-6 (Terzidou et al., 2006), H_2_S (Wang et al., 2019), and carotenoid (Avraham et al., 2019, 2021) induce OXTR expression. Among them, we were particularly interested in the estrogen system because the endogenous estrogen deficiency might desensitize predator odor exposure-induced short-term changes in cognitive behaviors and long-lasting changes in social behaviors (Gao et al., 2021). In addition, the orexin system plays a regulatory role in social or fear behavior (Faesel et al., 2021). In this study, the mRNA expressions of the ligands and receptors in the olfactory pathway [the olfactory bulb as the first olfactory center (e.g., Kobayakawa et al., 2007)], the amygdala for negative and attractive odor valence coding (Mori and Sakano, 2021), and the hypothalamus as an autonomic system and stress hormone control center (Onaka and Takayanagi, 2019) were measured to examine the association between olfactory behavior rescue and OXT–OXTR, estrogen, and orexin systems. In this study, repeated exposures to kairomone-containing coffee odor reportedly improved abnormal olfactory behaviors in heterozygous oxytocin receptor knock-in mice with an increase in Oxtr mRNAs in the olfactory bulb and amygdala.

## Materials and methods

### Experimental animals

Mice were cared for in accordance with the National Institutes of Health’s *Guide for the Care and Use of Laboratory Animals*. The Animal Ethics and Research Committee of Nihon University approved the experimental protocols prior to the start of this study.

Oxtr-Venus knock-in heterozygous OXTR (±) mice generated by Yoshida et al. (2017) were bred from heterozygous intercrosses. Only sexually inexperienced heterozygous OXTR (±) mice were used in this study. These mice were maintained on a mixed 129 × C57BL/6J genetic background. The animals were housed in groups of two to three animals in polycarbonate cages (26 × 18 × 12 cm) until the beginning of the behavioral experiments in a room at 22°C with a photoperiod of 12 h (non-reversed 12 h light/dark cycle) in a sterile animal facility. They were provided with *ad libitum* access to water and a standard murine diet (Lab Chow, CL-2, Clea-Japan, Tokyo, Japan). For the preference test for coffee odor, 11 wild-type OXTR (+/+) (*n* = 6 males, *n* = 5 females) and 10 heterozygous OXTR (±) (*n* = 5 males, *n* = 5 females) mice aged 8–10 weeks were used. For behavioral and molecular tests, 50 heterozygous OXTR (±) mice (*n* = 23 males, *n* = 27 females) aged 3–6 months were used.

### Coffee odor preference test in Y-maze

Eleven wild-type OXTR (+/+) and 10 heterozygous OXTR (±)mice were examined only in a single set of tests, conducted in a blinded manner using a custom-made Y-maze (long arm length: 450 mm, short-arm length: 400 mm, arm width: 100 mm) (Yamazaki et al., 1979; Yamaguchi et al., 1981; Osada et al., 2011). All animal behaviors were recorded with a video camera (IvisHF R42, Canon, Tokyo, Japan). In the acclimatization period, to avoid a neophobic response of mice to the coffee odor, mice were exposed to coffee odor once for 5 min in the home polycarbonate cage where a group of either two or three animals was housed. Before the experiment, mice were habituated to the Y-maze for 5 min. On the first experimental day, a polystyrene Petri dish (35-mm diameter; IWAKI, Japan) with 1 ml of water to serve as control was simultaneously inserted into both odor boxes connected to the short arms of the Y-maze. A mouse was placed at the end of the long arm. After opening the gate in front of the mouse, the investigation time in both the short arms was measured for 5 min (control test). On the next day, a Petri dish with 1 g of the grained coffee beans was placed on one of the odor boxes of the Y-maze, while another Petri dish with 1 ml of water was inserted into the other odor box (coffee odor preference test). The coffee odor was presented alternately from side to side in each odor box in each trial. Animals were placed on the long arm of the Y-maze, and then we measured the time spent, respectively, in each of the short arms based on where the snout oriented toward the odor source or water during the initial 5 min.

### Olfactory coffee odor stimuli

One gram of grained Sumatra dark roast coffee beans (Starbucks) was used as olfactory stimuli. A custom-made odor generator was prepared with a polystyrene Petri dish (35-mm diameter; IWAKI, Japan) with nine pores (5-mm diameter) at the bottom. To maintain coffee beans inside the odor generator and only let the odor through, a piece of porous paper was inserted between the bottom of the dish and the odorant. The odor generator injected with 1 ml of water was employed between non-exposure periods. These odor generators were placed on the center of the wire mesh lid of the polycarbonate home cage.

**FIGURE 1:**
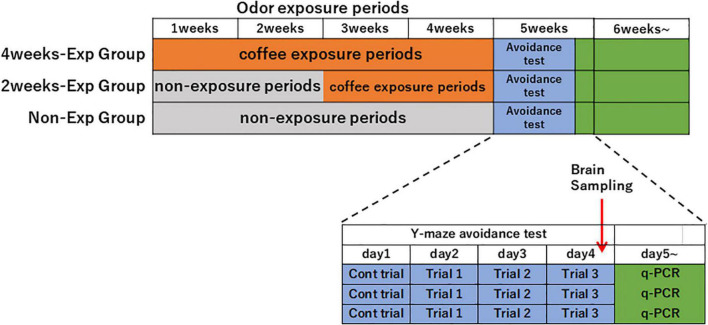
Time schedule of repeated coffee odor exposure experiments after 2 or 4 weeks in the heterozygous oxytocin receptor (OCTR) (±) mice. Heterozygous OXTR (±) mice were divided into non-exposure, 2-week, and 4-week groups and exposed to coffee odor. After the 4-week odor exposure period, a Y-maze avoidance test for butyric acid was conducted. After trial 3 of the avoidance tests, brain samples from all mice were collected.

Heterozygous OXTR (±) mice that were not used for the coffee odor preference tests were divided into three experimental groups: non-exposure (*n* = 17; eight males, nine females), 2-week (*n* = 16; seven males, nine females), 4-week exposure groups (*n* = 17; eight males, nine females) to coffee odor. Mice were exposed to the coffee or water odor for 2 h between 9:00 a.m. and 12:00 a.m., 5 days a week for 2 or 4 weeks (Figure 1).

### Unpleasant odor avoidance test for butyric acid in Y-maze

Butyric acid (Endres and Fendt, 2009; Utsugi et al., 2014; Osada et al., 2018) was used to generate avoidance of an unpleasant odor in each of the three groups of heterozygous OXTR (±) mice. These mice were examined only in a single set of avoidance tests, conducted in a blinded manner using a custom-made Y-maze. Before the experiment, mice were habituated to the Y-maze for 4 min. On the first experimental day, filter papers (2.5 × 2.5 cm) spotted with water (10 μl) to serve as controls were simultaneously inserted into both short arms of the Y-maze. The mouse was placed at the end of the long arm. After opening the gate in front of the mouse, the time spent in both short arms was measured for 4 min (control trial). On the next day, a filter paper (2.5 × 2.5 cm) spotted with 10 μl (56 μmol) of butyric acid was placed into the end of one of the short arms of the Y-maze, while another filter paper spotted with 10 μl of water was placed into the other short arm. The odor of butyric acid was presented alternately from side to side in each arm in each trial. Animals were placed on the long arm of the Y-maze, and then we measured the time spent, respectively, in each of the short arms, based on whether its snout was oriented toward the odor source or water, for 4 min (trial 1). This same protocol was repeated the next day (trial 2) and the day thereafter (trial 3). The avoidance rate was measured as the time spent in the short arm containing the control odor divided by the time spent in both short arms (control vs. butyric acid), multiplied by 100.

Each trial was conducted between 12:00 p.m. and 17:00 p.m. The floor of the test area was replaced with clean bench paper between trials to eliminate residual odor cues. All animal behaviors were recorded with a video camera (IvisHF R42, Canon, Tokyo, Japan).

### Real-time polymerase chain reaction

To determine changes in ligands and receptors of OXT, estrogen, and orexin systems, as well as stress responses to the repeated exposure to the coffee odor, real-time polymerase chain reaction (PCR) assays, were performed as follows: After trial 3, mice were anesthetized with gaseous isoflurane (Pfizer) and euthanized by cervical translocation. Thereafter, the dissected mouse brains were soaked in 5 ml of RNA later solution (Invitrogen) for 24 h at 4°C and preserved at –80°C. To collect mRNA from these samples, the preserved brains were defrosted at ambient temperature; thereafter, the brain samples were obtained in the same manner as previously described (Nishiuchi et al., 2018) and also confirmed by the mouse brain atlas (Franklin and Paxinos, 2008), that is, approximately 10 mg of the hypothalamus, olfactory bulb, and amygdala tissues were excised, placed in tubes, and homogenized by BioMasher Standard (Takara, Japan). The mRNA was extracted as follows: The mashed brain samples were used to purify RNAs using an RNeasy Plus Micro kit (Qiagen). cDNAs were generated by Reverse Transcription with the PrimeScript™ kit (Takara). To avoid genomic DNA contributing to the signal, we took the following measures: (1) RNA was treated in parallel in the presence and absence of reverse transcriptase, (2) primers were chosen to span ≥ 1 intron to distinguish PCR products from genomic DNA, and (3) after RNA purification, an RNase-free DNase (Qiagen) treatment was used to avoid genomic DNA contamination. Primer sequences for each PCR are shown in Table 1. Namely, Oxytocin receptor (*Oxtr*), oxytocin (*Oxt*), estrogen receptors α and β (*Er-*α, *Er-*β), estrogen synthetase (*Cyp*), orexin (*Orx*), orexin receptor1 (*Orx1r*), orexin receptor 2 (*Orx2r*), corticotropin-releasing hormone (*Crh*), and corticotropin-releasing hormone receptor (*Crhr*) for indicators of stress responses, and glyceraldehyde-3-phosphate dehydrogenase (GAPDH) were quantified. Real-time quantitative PCR reactions were performed using the following conditions: 94°C for 1 min (1 cycle), 94°C for 10 s, 62°C for 10 s, and 72°C for 10 s (40 cycles). GAPDH was used to normalize target mRNA expression (Yamamoto et al., 2013; Kakizaki et al., 2019). Real-time quantitative PCR reactions were performed with the Light cycler 96 system (Roche) using 20 μl of PCR solution samples containing Kapa SYBR^®^ FAST (Kapa; Salt River Cape Town, South Africa).

**TABLE 1:** Primer sequences used for RT-PCR and quantitative PCR.

Genes	Accession No.	Forward	Reverse	Region
*Oxtr*	NM_001081147	GCTGGACGCCTTTCTTCTT	TGAGAACAGCTCCTCTGACT	885—1180
*Oxt*	NM_011025	TGGATATGCGCAAGTGTCTC	GCGCTAAAGGTATTCCCAGAA	148— 448
*Crh*	NM_205769	TACCAAGGGAGGAGAAGAGAG	GCGCTAAAGGTATTCCCAGAA	214— 468
*Crhr*	NM_001313928.1	CTCCAGGGTTGTCTTCATCTAC	GCTAGTGAGCTTGCATCATTTC	1188—1482
*ERα*	NM_001302531	CCTGGACAGGAATCAAGGTAAA	GTGCCGGATATGGGAAAGAA	1447—1749
*ERβ*	NM_010157	TCTGGACACCTCTCTCCTTTAG	TTACGCCGGTTCTTGTCTATG	651—980
*Cyp*	NM_001348171	GAAGTGCCTGCAACTACTACA	GCCCGTCAGAGCTTTCATAA	320— 571
*ORXR1*	NM_001163027	ATAGCCTTGGTGGGCAATAC	AGCCTGGAGATAGGGAATGA	461—668
*ORXR2*	NM_001364551	CATGGCACCTCTGTGTCTTAT	GTGCTCGGATCTGCTTTATCT	815—1015
*Orexin*	NM_010410	GCCTCAGACTTCTTGGGTATTT	CGTGCAACAGTTCGTAGAGA	10— 251
*GAPDH*	NM_001289726	AACTTTGGCATTGTGGAAGG	ACACATTGGGGGTAGGAACA	585— 807

### Statistical analysis

Male and female data were combined and analyzed because no significant differences between sexes in behavioral assays have been observed previously (Osada et al., 2018). Data are presented as means ± SEM. A two-factor analysis of variance (two-factor ANOVA) was used to evaluate the main effect of brain area and mouse group plus the interaction effect. Bonferroni’s *post-hoc* test was used to assess the statistical significance of the differences between groups. Wilcoxon signed-rank test was used to assess the coffee odor preference test and the avoidance test of butyric acid odor, and two-factor repeated-measure ANOVA (two-factor RM-ANOVA) was used when appropriate. A *P*-value of < 0.05 was considered statistically significant.

## Results

### Coffee odor preference of wild-type OXTR (+/+) and heterozygous OXTR (±) mice in the Y-maze

**FIGURE 2:**
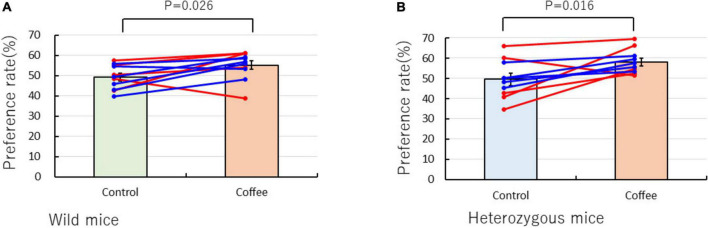
Y-maze preference test of wild-type oxytocin receptor (OXTR) (+/+) mice and heterozygous OXTR (±) mice to the coffee odor. The mean ± standard error preference rate (%) of the control (water vs. water) and coffee odor tests (water vs. grained coffee beans) were compared. **(A)** Wild-type mice (male = 6, female = 5) and **(B)** heterozygous mice (male = 5, female = 5) were used. The male and female data are described, respectively, in blue and red symbols and lines. The statistical significance of the differences between each of the samples was assessed by the Wilcoxon signed-rank test. An a-value of *P* < 0.05 was considered statistically significant.

A behavioral odor test was conducted in the Y-maze to determine whether the mice preferred or avoided the coffee odor. The preference rates of the mice over a 5-min period for the coffee odor are shown in Figures 2A, B. The Wilcoxon signed-rank test revealed significant differences between the control and coffee odor tests in the wild-type OXTR (+/+) (control test: 49.27 ± 1.81% vs. coffee preference test: 55.21 ± 2.03%, *P* = 0.026) and heterozygous OXTR (±) (control test: 49.56 ± 3.01% vs. coffee preference test: 58.17 ± 1.91%, *P* = 0.016) mice. Therefore, both kinds of mice were predisposed to be attracted to the coffee odor despite containing the kairomone compounds, instead of a kairomone-associated avoidance response (Figure 2).

### Avoidance of butyric acid odor by heterozygous OXTR (±) mice in the Y-maze

To determine whether the repeated coffee odor exposure rescues the low reactivity of heterozygous OXTR (±) mice to unpleasant odors at the first presentation in non-social and non-context conditions, we performed a Y-maze avoidance test. We examined the avoidance rates of the three different exposure groups of heterozygous OXTR (±) mice to butyric acid. The behavioral patterns of the non-exposure, 2-week, and 4-week exposure heterozygous OXTR (±) mice groups were compared with previously reported data on non-exposed wild-type OXTR (+/+) mice (*n* = 16) (Osada et al., 2018). We found higher avoidance rates for trial 1 than for the control trial and trial 3.

**FIGURE 3:**
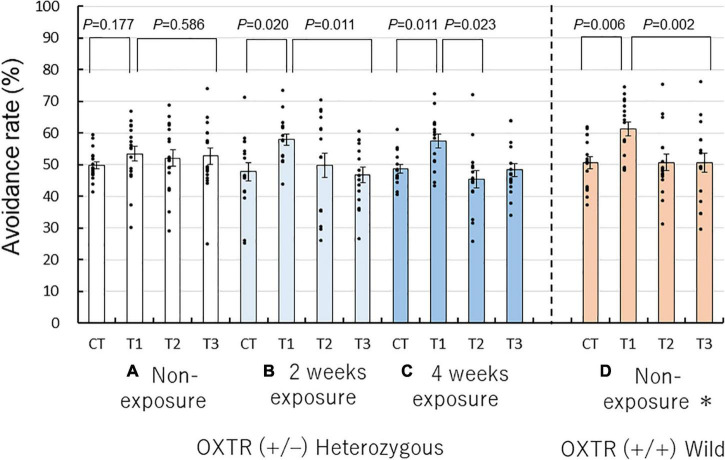
Repeated coffee odor exposure-induced rescue of abnormal olfactory behaviors of heterozygous oxytocin receptor (OXTR) (±) mice. Avoidance rates of non- **(A)**, 2-week **(B)**, 4-week exposures to coffee odor of heterozygous OXTR (±) mice **(C)**, and non-exposure wild-type mice **(D)** (*n* = 16) (Osada et al., 2018). Black dots describe individual data. Non-exposure heterozygous OXTR (±) mice exhibited an abnormal avoidance behavior to the unpleasant odor on the first trial [trial 1 in **(A)**], compared with non-exposure wild-type mice **(D)**. This abnormal avoidance behavior was rescued by 2 or 4 weeks of repeated exposure to coffee odor **(C,D)**. The statistical significance of the differences in the avoidance rates of the four groups was assessed using the Wilcoxon signed-rank test. Avoidance rates marked by the letter a are significantly different from those marked by the letter b (*P* < 0.05).

The Wilcoxon signed-rank test revealed that trial 1 was significantly higher in avoidance rate compared with the control trial and trial 3 in the 2- and 4-week coffee odor exposure heterozygous OXTR (±) mice and non-exposure OXTR (+/+) wild mice, but not the non-exposure heterozygous OXTR (±) mice (Figures 3A–C).

In the 2-week exposure group, the avoidance rates were significantly higher for trial 1 than for the control trial (57.95 ± 1.92% vs. 47.79 ± 3.03%; *P* = 0.020, Wilcoxon signed-rank test, Figure 3B) was higher for trial 1 than for the trial 3 (57.95 ± 1.92% vs. 46.85 ± 2.47%; *P* = 0.011, Figure 3B). Similarly, in the 4-week exposure group, there were significant differences between trial 1 and the control trial (57.47 ± 2.22% vs. 48.68 ± 1.41%; *P* = 0.011, Figure 3C). In contrast, in the non-exposure heterozygous OXTR (±) group, there were no significant differences between trial 1 and the control trial (53.45 ± 2.35% vs. 49.84 ± 1.18%, *P* = 0.177, Wilcoxon signed-rank test, Figure 3A) and between trial 1 and trial 3 (53.45 ± 2.35% vs. 52.73 ± 2.47%, *P* = 0.586, Figure 3A). In non-exposed wild-type mice, there were significant differences between trial 1 and the control trial (61.29 ± 2.26% vs. 50.63 ± 1.93%, *P* = 0.006, Figure 3D), which were similar to those of the 2- and 4-week exposure heterozygous OXTR (±) mice.

When compared with the avoidance rates of the control trial, wild-type and 2- and 4-week exposure heterozygous OXTR (±) mice significantly avoided the butyric acid odorant in trial 1, but the non-exposed heterozygous OXTR (±) mice did not markedly change the avoidance rate (Wilcoxon signed-rank test for changes in avoidance rates, *P* = 0.177). In other words, when exposed to coffee odor, heterozygous OXTR (±) mice performed such as wild-type mice in terms of avoidance behavior to butyric acid on the first encounter; however, non-exposed heterozygous OXTR (±) mice did not show such avoidance behavior. The avoidance behavior observed in trial 1 may mean that the coffee odor exposure rescued the innate avoidance response of heterozygous OXTR (±) mice to butyric acid.

Similar to wild-type mice, the 4-week coffee odor exposure mice became habituated (as expected by chance in selecting the short arm scented with the target odor) to the unpleasant odor of butyric acid in trials 2 and 3, whereas non-exposed heterozygous OXTR (±) mice maintained a slightly higher avoidance rate than due to chance (Figure 3A). Interestingly, the 2-week coffee exposure group appeared to be habituated in trial 2 but less than that of the 4-week coffee odor exposure mice. This minor difference will be investigated in future studies.

As previously observed in wild-type and non-exposure heterozygous mice (Osada et al., 2018), the repeated coffee odor exposure’s main effect was confirmed on the avoidance rates among trials (*F*(3, 162) = 10.657, *P* = 0.0001, two-factor RM-ANOVA) but not between sexes [*F*(1, 54) = 0.263, *P* = 0.610], with no interaction between trial and sexes [*F*(3, 162) = 0.519, *P* = 0.586] and between trial and four mouse exposure groups [*F*(9, 162) = 1.175, *P* = 0.314]. The exposure groups with combined male and female data were compared because there were no marked differences between the sexes and trials.

**FIGURE 4:**
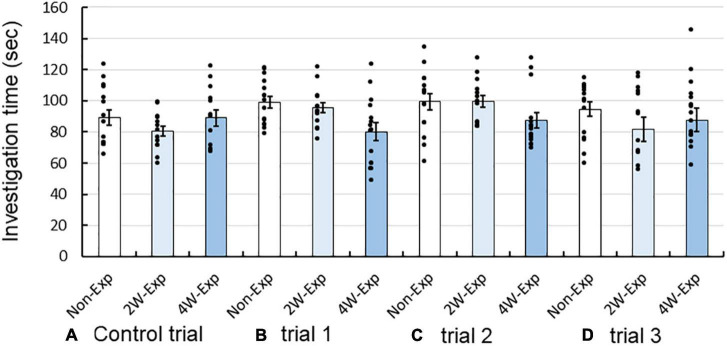
Changes in investigation time of heterozygous oxytocin receptor (OXTR) (±) mice after 2 or 4 weeks of repeated exposure to coffee odor. Investigation time of the non-exposure, 2-week, and 4-week exposure groups in the **(A)** control trial, **(B)** trial 1, **(C)** trial 2, and **(D)** trial 3. Black dots describe individual data. The statistical significance of the differences in the investigation time between the three groups was assessed using two-factor repeated-measure ANOVA followed by Bonferroni’s *post-hoc* tests.

Next, we compared the total investigation time in both the short arms of the Y-maze between the non-exposure, 2-week, and 4-week exposure groups. However, there were no marked differences between the groups in the control trial and trials 1–3 (Figure 4). These data suggested that coffee odor exposure did not affect odor investigation behaviors of heterozygous OXTR (±) mice. Two-factor RM-ANOVA revealed that the repeated coffee odor exposure had no major effect on the investigation time among trials [*F*(3, 123) = 1.935, *P* = 0.127, two-factor RM-ANOVA], no interaction between trial and sexes (*P* = 0.655), but revealed interactions between trial and mouse exposure groups [*F*(9, 123) = 2.229, *P* = 0.045]. There were no marked differences between exposure groups in each of the trials (Bonferroni *post-hoc* test).

### mRNA expression levels of *Oxtr* and other neuropeptides or receptors in the coffee odor-exposed heterozygous OXTR (±) mouse brain

**FIGURE 5:**
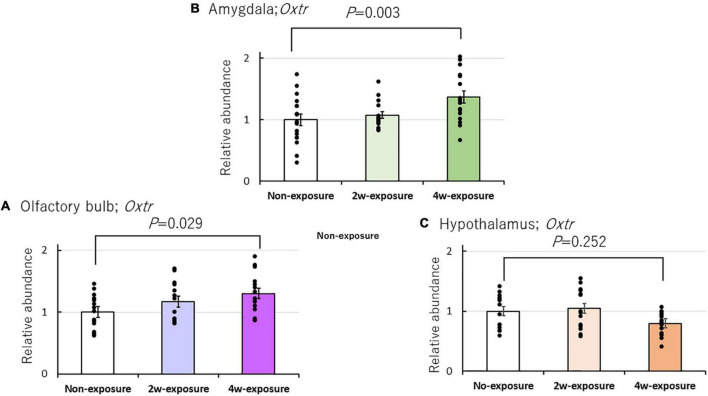
Effects of repeated coffee odor exposure on *Oxtr* mRNA expression in the three brain areas. Effects of odor stimulation on *Oxtr* mRNA expression in the olfactory bulb **(A)**, the amygdala **(B)**, and the hypothalamus **(C)**. Black dots describe individual data. The bar graphs show the mean ± SE. The statistical significance (*P* < 0.05) of the difference between genes in the non-exposure, 2-week, or 4-week exposure groups was tested by two-factor ANOVA followed by a Bonferroni *post-hoc* test.

To examine the effects of repeated coffee odor exposure on *Oxtr* mRNA expression in the brain of heterozygous OXTR (±) mice, we performed RT-PCR of three brain regions, namely, the olfactory bulb, the hypothalamus, and the amygdala. *Oxtr* expression increased in the olfactory bulb and amygdala of heterozygous OXTR (±) mice after 4 weeks of exposure to coffee odor. Two-factor ANOVA revealed the main effect between the three brain areas [*F*(2, 133) = 6.404, *P* = 0.002], but not the main effect of the coffee exposure durations [*F*(2, 133) = 2.866, *P* = 0.061], and revealed the interaction between three brain area and coffee exposure durations [*F*(4, 133) = 4.630, *P* = 0.002]. Changes in *Oxtr* expression were normalized by the local expression of GAPDH. A Bonferroni test indicated significantly increased *Oxtr* expression in the olfactory bulb (*P* = 0.029) of 4-week coffee exposure OXTR (±) mice compared with non-exposure OXTR (±) mice and in the amygdala (*P* = 0.003) of 4-week coffee exposure groups to non-exposure OXTR (±) mice (Figures 5A, B). In contrast, *Oxtr* expression did not markedly change in the hypothalamus after 4 weeks of coffee odor exposure in OXTR (±) mice (Figure 5C).

**FIGURE 6:**
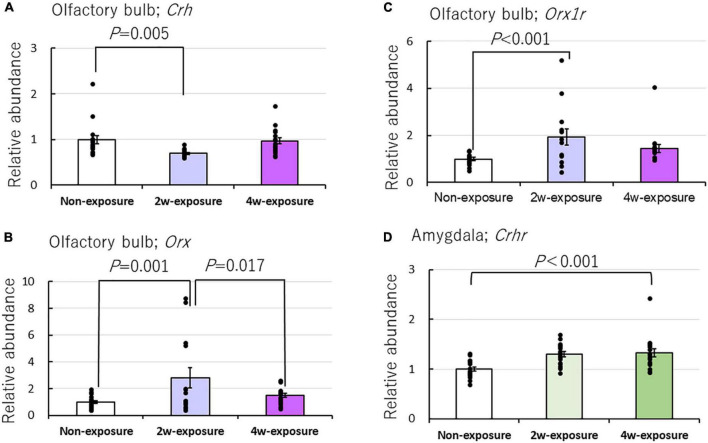
Effects of repeated coffee odor exposure on gene expression in the olfactory bulb and amygdala. Effect of odor stimulation on the mRNA expression of **(A)** *Crh*, **(B)** *Orx*, **(C)** *Orx1r* in the olfactory bulb, and **(D)** *Crhr* in the amygdala. Black dots describe individual data. Data show the mean ± standard error (SE). The statistical significance of the difference between genes in the non-exposure, 2-week, or 4-week exposure groups was tested by two-factor ANOVA followed by a Bonferroni *post-hoc* test. A *P*-value of < 0.05 was considered statistically significant.

*Oxt, Crh, Crhr, Er-*α, *Er-*β, *Cyp, Orx, Orx1r*, and *Orx2r* mRNA expressions were in addition to quantified. The following results were obtained from this study: *Crh:* brain area main effect [*F*(2, 139) = 1.188, *P* = 0.308, two-factor ANOVA], brain area by coffee exposure durations interaction [*F*(4, 139) = 2.861, *P* = 0.044*, the asterisk (*) was used to denote the significant difference with *P* < 0.05]; *Crhr:* brain area main effect [*F*(2, 139) = 19.978, *P* = 0.0001*] and brain area by coffee exposure durations interaction [*F*(4, 139) = 5.371, *P* = 0.0001*]; *Orx:* brain area main effect [*F*(2, 132) = 8.558, *P* = 0.0001*] and brain area by mouse group interaction [*F*(4, 132) = 3.177, *P* = 0.016*]; and *Orx1r:* brain area main effect [*F*(2, 133) = 8.807, *P* = 0.0001*] and brain area by mouse group interaction [*F*(4, 133) = 3.502, *P* = 0.009*]. Bonferroni test indicated that after 2 weeks of exposure to the coffee odor, *Crh* mRNA did not increase but significantly decreased in the olfactory bulb of OXTR (±) mice compared with that of non-exposed OXTR (±) mice (*P* = 0.005). However, it significantly increased *Orx* (*P* = 0.001) and *Orx1r* expressions (*P* < 0.001) compared with that of non-exposed OXTR (±) mice (Figures 6A–C and Table 2).

**TABLE 2:** Effects of coffee odor on gene expression in heterozygous OXTR (±) mice.

Relative abundance (Target gene / GAPDH;mean ± SEM)				
		Non-exposure	2 weeks	4 weeks
Oxtr	Olfacory bulb	1.00 ± 0.07^a^	1.17 ± 0.09	1.30 ± 0.08^b^
	Amygdala	1.00 ± 0.10^a^	1.07 ± 0.05	1.37 ± 0.10^b^
Oxt	Hypothalamus	1.00 ± 0.08^a^	1.05 ± 0.09	0.80 ± 0.05^a^
	Olfacory bulb	1.00 ± 0.19	0.83 ± 0.09	0.95 ± 0.16
	Amygdala	1.00 ± 0.30	1.89 ± 0.61	0.66 ± 0.22
	Hypothalamus	1.00 ± 0.10	0.81 ± 0.14	0.84 ± 0.11
Crh	Olfacory bulb	1.00 ± 0.09^a^	0.70 ± 0.02^b^	0.96 ± 0.07
	Amygdala	1.00 ± 0.07	0.94 ± 0.04	0.96 ± 0.07
Crhr	Hypothalamus	1.00 ± 0.06	0.98 ± 0.07	0.86 ± 0.07
	Olfacory bulb	1.00 ± 0.04	0.92 ± 0.03	0.98 ± 0.02
	Amygdala	1.00 ± 0.04^a^	1.30 ± 0.06^b^	1.32 ± 0.08^b^
Erα	Hypothalamus	1.00 ± 0.06	0.89 ± 0.05	1.03 ± 0.06
	Olfacory bulb	1.00 ± 0.13	1.02 ± 0.08	0.90 ± 0.06
	Amygdala	1.00 ± 0.10	1.08 ± 0.12	1.19 ± 0.10
Erβ	Hypothalamus	1.00 ± 0.11	0.88 ± 0.08	0.68 ± 0.08
	Olfacory bulb	1.00 ± 0.08	0.78 ± 0.10	0.99 ± 0.09
	Amygdala	1.00 ± 0.10	1.05 ± 0.12	1.08 ± 0.09
Cyp	Hypothalamus	1.00 ± 0.04	0.98 ± 0.07	0.82 ± 0.06
	Olfacory bulb	1.00 ± 0.29	0.93 ± 0.21	1.21 ± 0.18
	Amygdala	1.00 ± 0.26	0.97 ± 0.23	1.40 ± 0.36
Orx1R	Hypothalamus	1.00 ± 0.14	0.91 ± 0.11	1.00 ± 0.13
	Olfacory bulb	1.00 ± 0.06^a^	1.92 ± 0.35^b^	1.44 ± 0.18
	Amygdala	1.00 ± 0.12	0.96 ± 0.03	0.84 ± 0.06
Orx2R	Hypothalamus	1.00 ± 0.06	0.98 ± 0.06	0.86 ± 0.06
	Olfacory bulb	1.00 ± 0.14	0.91 ± 0.09	1.03 ± 0.15
	Amygdala	1.00 ± 0.10	0.85 ± 0.04	0.88 ± 0.05
Orexin	Hypothalamus	1.00 ± 0.06	0.89 ± 0.04	0.79 ± 0.08
	Olfacory bulb	1.00 ± 0.11^a^	2.78 ± 0.76^b^	1.48 ± 0.15^a^
	Amygdala	1.00 ± 0.11^a^	1.31 ± 0.10	0.68 ± 0.09
	Hypothalamus	1.00 ± 0.08	0.86 ± 0.09	0.79 ± 0.05

The differences between three experimental groups was assessed by two-factor ANOVA followed by Bonferroni *post-hoc* test. Different letters between the groups indicate a significant difference (*P* < 0.05).

In addition, the Bonferroni test indicated significantly increased *Crhr* expression (*P* < 0.001) in the amygdala of the coffee-exposed heterozygous OXTR (±) mice compared with non-exposure OXTR (±) mice (Figure 6D). Increased *Crhr* expression in the amygdala and increased Oxtr expressions in the olfactory bulb and the amygdala might rescue the abnormal olfactory behaviors in heterozygous OXTR (±) mice. *Oxt, Er-*α, and *Er-*β, regulators of the gonadotropic hormone system, were not markedly affected by repeated exposure to coffee odor (Table 2).

## Discussion

We first attempted to rescue a heterozygous OXTR (±) mice with an acute olfactory response dysfunction to an unpleasant odor with 2–4 weeks of repeated exposure of the heterozygous OXTR (±) mice to a preferable food odor. Abnormal olfactory behaviors in heterozygous oxytocin receptor knock-in mice successfully improved with repeated exposure to kairomone-containing coffee odor by an increase in *Oxtr* mRNAs in the olfactory bulb and the amygdala. This repeated coffee odor exposure-induced improvement in atypical olfactory behavior responses to the unpleasant odor in heterozygous OXTR (±) mice as a model of mild cases of ASD could support clinical trials of coffee odor exposure therapy for some of the mild cases of ASD. Further discussions on our results and limitations are presented later.

### Wild-type and heterozygous mice prefer the coffee odor

The three kairomone components of the coffee odor were alkyl pyrazine analogs: 2,6-dimethylpyrazine, 2,3,5-trimethylpyrazine, and 3-ethyl-2,5-dimethylpyrazine (Osada et al., 2013). As previously reported (Tabuchi et al., 1991; Yamato et al., 2002; Seo et al., 2008; Matsukawa et al., 2011; Ohata et al., 2020; Nishi et al., 2022), the wild-type OXTR (+/+) and heterozygous OXTR (±) mice exhibited a preference to the coffee odor (Figures 2A, B) despite containing the three kairomone compounds emitted from the common gray wolf, instead of kairomone-associated avoidance or stress responses. In fact, the coffee odor contains many pleasant odors such as nuts or cheese, and stress-induced *Crh* mRNA did not increase, but transiently decreased in the olfactory bulb of the coffee odor-exposed OXTR (±) mice (Table 2). This result is consistent with the observation that the mixture of the predator TMT odor and rose odor counteract the TMT odor-induced stress response in the plasma adrenocorticotrophic hormone by suppressed responses in the anterior piriform cortex and the bed nucleus of the stria terminalis (Matsukawa et al., 2011). However, it is yet to be determined which pleasant coffee bean compounds would be the key determinant for inducing the positive coffee odor valence in mice. A possible antagonistic effect between the three kairomone compounds and some pleasant compounds, such as 2-methylbutanal (almond), 2,4,5-trymethyl-1,3-oxazole (aged cheeses), heptan-2-ol (coconut), 2,5-dimethylpyrazine (roasted nut), 5-methylfuran-2-carbaldehyde (almond, caramel), octanoic acid (cheese), 2-methylsulfanylfuran (coffee), and others (Mahmud et al., 2022) remains unresolved. Repeated exposure to such a mixed odor of antagonistic kairomone and pleasant food compounds would result in the increase of specific receptor cells in mice in a lineage-specific manner, as observed in the increased peripheral olfactory sensitivity to the same odorant but not the other odor in the anosmic mouse strain for androsterone or isovaleric acid (Wang et al., 1993).

### Repeated exposure to coffee odor improved the abnormal olfactory behavior of the heterozygous mice with accompanying increases in the OXTR expression in the olfactory bulb and the amygdala

Here, repeated coffee odor exposure was found to increase *Oxtr* expression in the olfactory bulb and amygdala (the olfactory pathway) but not in the hypothalamus (not the olfactory pathway) of heterozygous OXTR (±) mice, leading to a remarkable abnormal olfactory behavior rescue; the impaired olfactory avoidance from an unpleasant odor at the first presentation. It is possible that local estrogen would mediate the observed increases in *Oxtr* expression (Choleris et al., 2003, 2012). However, our real-time PCR data indicated no marked increase in the Er-α and β mRNA expressions and estrogen synthetase (*Cyp*) in the olfactory bulb and amygdala of the coffee odor-exposed mice (Table 2), suggesting no estrogen system associated with the observed olfactory behavior rescue. OXTR expression changes in the other olfactory and non-olfactory areas, as well as the association between the observed rescue of the abnormal olfactory behavior and the other OXTR expression inducers, are unknown, which should be examined for a better understanding of the coffee odor-induced rescue of the acute odor avoidance behavior in future studies.

Previously, two aspects of abnormal olfactory behaviors of homozygous OXTR (-/-) and heterozygous OXTR (±) mice (Osada et al., 2018) have been observed. Homozygous and heterozygous OXTR (±) mice failed to avoid this odor at the first presentation (trial 1) in contrast to the significant avoidance of wild-type mice of the butyric acid odor, suggesting that reduced *Oxtr* expression in these mice would be attributable to the impaired acute olfactory avoidance behavior. In addition, dissimilar to the habituation of wild-type mice to the butyric acid odor (trials 2 and 3), the weak aversive responses of heterozygous OXTR (±) mice scarcely changed, and homozygous OXTR (-/-) mice exhibited enhanced avoidance (Osada et al., 2018). Previous reports also demonstrated that heterozygous OXTR (±) mice show abnormal body temperature control and social, feeding, contextual, and non-contextual olfactory behaviors (Takayanagi et al., 2005; Kasahara et al., 2013; Sala et al., 2013; Oettl et al., 2016; Osada et al., 2018). Therefore, these three types of OXTR-deficient mice demonstrated that distinct degrees of OXTR deficiency could induce distinct abnormal olfactory behavior phenotypes. Further analyses of the three OXTR-deficient mice types at the molecular and behavior levels could elucidate how the OXT and associated signaling in the olfactory and non-olfactory pathways (Igarashi et al., 2014; Dampney, 2015, 2016; Isosaka et al., 2015; Miyazono et al., 2018; Mori and Sakano, 2021) to control various olfactory behaviors and how the repeated exposure to coffee odor rescues OXTR deficiency-induced abnormal olfactory and non-olfactory behaviors. This study is the first to successfully test a coffee odor therapy for the hyposensitive acute avoidance behavior to the unpleasant odor in heterozygous OXTR (±) mice as a model of mild ASD cases. The systematic data and identified similarities collected between various patients with ASD and model mice will enable clinical trials to be conducted with coffee odor therapy for patients with mild ASD in the future.

### Transient change of CRH, ORX, and ORX1R expression in the olfactory bulb

*Crh* is expressed throughout the mice’s brains. The highest density of *Crh* neurons is found in the main olfactory bulb and hypothalamic paraventricular nucleus (PVN) (Kondoh et al., 2016; Peng et al., 2017). Stress induces CRH production and releases in the PVN, activating the hypothalamus–pituitary gland–adrenal gland axis (HPA) (Vale et al., 1981). As described earlier, *Crh* expression in the hypothalamus was not affected by repeated coffee exposure (Table 2).

The observed minor increase in *Oxtr* gene expression in the olfactory bulb would explain a decrease in *Crh* gene expression in heterozygous mice exposed to coffee odor for 2 weeks. The inhibitory effect of OXT on *Crh* gene expression in rodent PVN and neuroblastoma cells expressing *Oxtr* supports this observation (Jurek et al., 2015). *Crh* interneurons (Huang et al., 2013) are also associated with mitral cells in the external plexiform layer of the olfactory bulb. Conversely, the corticotropin-releasing hormone-binding protein (CRHBP), a stress hormone CRH antagonist, is specifically expressed in OXTR interneurons and has an anxiolytic effect (Li et al., 2016). Therefore, increased *Oxtr* expression attenuates the stress response in the PVN and/or CRH expression at the interneurons in the olfactory bulb. ORX and OrxR1 are also notably increased in the olfactory bulb (Figure 6), suggesting a reciprocal relationship between the orexin and CRH systems (Summers et al., 2020). The increased ORX and OXT can stimulate ORX neurons in mice (Tsujino et al., 2005). Increased ORX-enhanced lateral inhibition from gannma amino butyric acid agonistic (GABAergic) interneurons, which express ORX receptors (Hardy et al., 2005), may modulate the olfactory signal processing in the mitral cells and the resultant behaviors in addition to this ORX neuron-mediated odor signaling modification.

### The change of OXTR expression in the amygdala

Increases in *Oxtr* and *Crhr* expression were observed in the amygdala (Table 2 and Figure 6B). The amygdala is involved in inducing negative and positive valences from sensory input (Janak and Tye, 2015). Specifically, odor signals are transmitted to the cortical amygdaloid nucleus and the medial amygdaloid nucleus (Price, 2003; Matsumoto et al., 2009). In particular, some serotonin 2A receptor-expressing cells in the central amygdala act as a hierarchy generator between the negative valence of innate and learned odor information (Isosaka et al., 2015). In addition, the amygdalo-piriform transition area contains neurons upstream of CRH neurons, which are activated by volatile predator odors and cause an increase in plasma adrenocorticotropic hormone as make fear stress responses (Kondoh et al., 2016). Furthermore, OXTR is highly expressed in serotonin neurons in the amygdala nuclei (Yoshida et al., 2009). These observations suggest a possibility that increased *Oxtr* expression may enhance serotonin-expressing neuron activities that rescue valence sensitivities to unpleasant and pleasant odors. However, the increased *Crhr* expression effect is yet unknown, and further research is required.

## Conclusion

The present study revealed that repeated coffee odor exposure rescues olfactory behaviors of abnormal heterozygous OXTR (±) mice as a model of mild cases of ASD and, probably, with the response to the CRH and ORX systems by an increase in *Oxtr* expression in the olfactory bulb and the amygdala. However, future studies are needed to confirm whether the coffee and another odor or some key compounds could improve the social behavior abnormality, likely ASD and other mental disorders induced by OXT–OXTR system dysfunction and other neural peptide systems through what molecular mechanisms in which olfactory signaling pathway.

## Data availability statement

The original contributions presented in this study are included in the article/supplementary material, further inquiries can be directed to the corresponding author.

## Ethics statement

The animal study was reviewed and approved by the Animal Ethics and Research Committee of the Nihon University.

## Author contributions

KO, TO, and AH conceived and designed the experiments. KN developed the OXTR-Venus knock-in mice. RK, MT, and KO conducted the experiments. MO and KO drafted the manuscript. All authors contributed to the article and approved the submitted version.
